# Indoor incense burning and impaired lung function in patients with diabetes

**DOI:** 10.1038/s41598-024-84565-z

**Published:** 2025-01-04

**Authors:** Yaxian Meng, Xiaojie Han, Chao Yi, Miao Liu, Ruoqing Chen, Haitao Chen, Tao Zhou, Jianjun Liu, Xiaoliang Chen, Yiqiang Zhan

**Affiliations:** 1 Department of Epidemiology, School of Public Health (Shenzhen), Sun Yat-Sen University, Shenzhen, China; 2 Guangming Center for Disease Control and Prevention, Shenzhen, China; 3 Shenzhen Center for Disease Control and Prevention, Shenzhen, China; 4 Institute of Environmental Medicine, Karolinska Institutet, Stockholm, Sweden

**Keywords:** Incense, Spirometry, Impaired lung function, Diabetes, Gender-disparity, Epidemiology, Environmental social sciences, Risk factors, Environmental impact

## Abstract

While recent studies have indicated a potential link between incense burning and respiratory diseases, there is a lack of data specifically focused on diabetic patients. To explore the relationship between indoor incense burning and impaired lung function among Chinese individuals with diabetes, a comprehensive cross-sectional study was undertaken, enrolling 431 adults diagnosed with diabetes. Information on incense burning and characteristics was collected using a structured questionnaire. The outcome of the study, impaired lung function, was assessed using spirometry. Multivariable logistic regression models were employed. In the fully adjusted model, participants exposed to indoor incense burning exhibited 130% higher odds of impaired lung function compared to those not exposed, as indicated by an odds ratio (OR) of 2.3 (95% confidence interval [CI]: 0.97, 5.16; *P* = 0.05). Notably, this association was statistically significant only in men (OR = 3.39; 95%CI: 1.07, 9.82; *P* = 0.03). Our study has elucidated an association between exposure to indoor incense burning and impaired lung function in individuals with diabetes, independently of demographic factors. These findings underscore the importance of considering indoor environmental factors, such as incense burning, in the comprehensive management and care of diabetic individuals.

## Introduction

Diabetes mellitus, a prevalent metabolic disorder, has experienced a substantial increase in the past three decades, primarily due to lifestyle changes, such as poor diet, physical inactivity, and rising obesity rates^1^. Research indicates that diabetic patients frequently manifest impaired lung function, characterized by reduced forced vital capacity (FVC) and forced expiratory volume in one second (FEV_1_)^2–6^. These impairments could potentially lead to diminished quality of life, decreased exercise capacity^7^, poorer clinical outcomes, heightened risk of deterioration and mortality, and increased socioeconomic burden^8–10^ when compared to individuals with normal lung function. Consequently, the proactive monitoring of lung function, identification of related risk factors, and early intervention and management strategies are crucial for potentially enhancing the quality of life for individuals with diabetes.

Burning incense is a traditional ritual that is widely practiced across various communities in Asia and Arabian regions^11,12^. However, the act of burning incense has the capacity to produce significant quantities of particles, aerosols, as well as gaseous pollutants, alongside a variety of volatile organic compounds, all of which have potential to induce detrimental impacts on people’s health^13–15^. Recent studies have suggested a potential correlation between indoor incense burning and respiratory diseases^13,16–20^, while some studies have found no substantial association^11,21^. Interestingly, a few studies even propose that burning incense could mitigate the risk of asthma^22^. Given these conflicting findings, the relationship between incense burning and respiratory disease risk is still unclear, highlighting the need for further investigation to clarify these associations.

The objective of this study is to investigate the association of indoor incense burning with lung function among patients with diabetes. Building upon existing studies, the hypothesis posited is that diabetics exposed to indoor incense burning are at a higher risk of impaired lung function compared to non-exposed individuals. By establishing a connection between indoor incense burning and impaired lung function, particularly in diabetic individuals, this study aims to offer valuable insights that could enhance the management and intervention strategies for improving the respiratory health of diabetic patients.

## Methods

### Study population

**Fig. 1:**
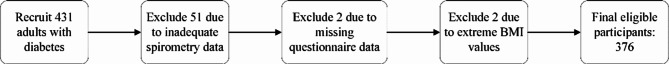
Flowchart of participant selection.

Study participants were recruited in Shenzhen, China between June and July 2023. A two-stage sampling method was employed to enroll participants. Initially, four community health service centers were randomly chosen. Subsequently, individuals with diabetes were randomly invited through phone calls, text messages, and WeChat. Enrolment included face-to-face interviews, physical examinations, and spirometry tests. The inclusion criteria were: age ≥ 18 years, diagnosed and registered diabetes, and no contraindications to spirometry. Exclusion criteria encompassed refusal to undergo spirometry, poor quality spirometry results, incomplete questionnaire data, and missing height or weight information. A total of 431 eligible adults with diabetes were recruited, of which 51 were excluded due to inadequate spirometry data (unacceptable quality spirometry = 43, refusal to undergo testing = 8), two were excluded due to missing questionnaire data, and two were excluded due to extreme body mass index values. Ultimately, the final study analytical sample comprised 376 individuals, accounting for 87.2% of the initial sample. The flowchart of participant selection is presented in Fig. 1. The Ethical Review Committee at the School of Public Health (Shenzhen), Sun Yat-Sen University approved the study, and all participants provided informed consent at recruitment. Additionally, we confirm that all methods were performed in accordance with relevant guidelines and regulations.

### Exposure assessment

Information on indoor incense burning was collected by a structured questionnaire and all participants were guided by trained interviewers to ensure a consistent understanding of the question. Participants who answered “*yes*” to the question “*Do you burn incense indoors in the past year?*” were classified as the exposed group. Participants who answered “*no*” to the question were classified as the non-exposed group.

### Impaired lung function measurements

Spirometry was conducted using the nddEasyOne portable lung function instrument (AG, NDD, Switzerland) and Easy on-PC software. Participants were introduced to the procedure and guided to practice the required movements to quickly familiarize themselves with the process. All measurements were performed with participants standing upright and utilizing a disposable mouthpiece. A minimum of three measurements were obtained from each participant, ensuring the presence of at least three acceptable trials and a within-trial difference of ≤ 150 mL between the two highest values of FEV_1_ and FVC. The best result from at least three technically satisfactory measurements was recorded. Predicted FVC values were calculated using previously published equations^23^, taking into account participants’ age, gender, height, and race/ethnicity. Impaired lung function was defined as individuals reporting an FVC < 80% of the predicted FVC value, an FEV_1_/FVC ratio < 0.7, or both of these criteria^24,25^.

### Potential confounders

In this analysis, we considered several demographic variables, including age, gender, body mass index (BMI), educational attainment, and smoking status, as potential confounders. Age was determined by calculating the time difference between the birth date and interview date. Educational attainment was categorized into three groups: primary school or below, junior high school, and high school or above. Smoking status was classified as current smoker, never smoked, or ever smoker. Height and weight measurements were taken while participants were wearing lightweight clothing and not wearing shoes. The BMI was calculated as the weight in kilograms divided by the square of the height in meters (kg/m^2^).

### Statistical analysis

Descriptive statistics, such as frequency, percentage, means, and standard deviations (SD), were calculated and cross-tabulated by gender to provide a comprehensive overview of the data distribution. Group comparisons were conducted using the Chi-square (χ2) test for categorical variables and one-way analysis of variance(ANOVA) for continuous variables, enabling a robust assessment of the differences among the study groups.

To explore the association of indoor incense burning with impaired lung function, multivariable logistic regression models were employed. The odds ratios (OR) and corresponding 95% confidence intervals (CI) were reported with the non-exposed group serving as the reference. These models were adjusted for various potential confounders: model 1 included gender and age; model 2 incorporated additional adjustment for BMI; and model 3 included adjustments for educational attainment, and smoking status, expanding upon the adjustments made in model 2. Recognizing the differences in characteristics between genders, gender-specific analyses were carried out separately for men and women, allowing for a more nuanced understanding of the potential effects of indoor incense burning on lung function within each gender group.

All statistical analyses were conducted using RStudio (2024.09.0 + 375 “Cranberry Hibiscus” Release, https://posit.co/download/rstudio-desktop/). A two-tailed p-value of < 0.05 was considered statistically significant.

## Results

Table 1 presents the characteristics of the study population. Out of 376 participants, 222 (59.0%) were men, and 154 (41.0%) were women. The mean age of all participants was 53.1 ± 9.6 years, with women having a higher mean age (55.0 ± 10.3 years) compared to men (51.7 ± 8.9 years). The mean BMI for all participants was 25.5 ± 3.8 kg/m^2^, with similar values observed for men and women at 25.5 ± 3.2 kg/m^2^ and 25.6 ± 4.5 kg/m^2^, respectively. Additionally, 121 (32.2%) had a high school diploma or higher, 80 (21.3%) were active smokers, and 35 (9.1%) were exposed to indoor incense burning. Notably, a higher proportion of men engaged in smoking and had attained higher levels of education.

**Table 1:** Socio-demographical characteristics of study participants.

Characteristics	Overall (*n* = 376)	Men (*n* = 222)	Women (*n* = 154)	*P*
Mean age, mean(SD, years)	53.1 (9.6)	51.7 (8.9)	55 (10.3)	0.001***
BMI, mean(SD, kg/m^2^)	25.5 (3.8)	25.5 (3.2)	25.6 (4.5)	0.65
Educational attainment, n(%)				< 0.001***
Primary school or below	117 (31.1)	35 (15.8)	82 (53.2)	
Junior high school	138 (36.7)	89 (40.1)	49 (31.8)	
High school or above	121 (32.2)	98 (44.1)	23 (14.9)	
Smoke status, n(%)				< 0.001***
Active smoker	80 (21.3)	79 (35.6)	1 (0.6)	
Never smoked	246 (65.4)	96 (43.2)	150 (97.4)	
Ever smoker	50 (13.3)	47 (21.2)	3 (1.9)	
Incense burning, n(%)				0.81
Yes	35 (9.1)	20 (9)	15 (9.7)	
No	341 (90.9)	202 (91)	139 (90.3)	

BMI, body mass index; SD, standard deviation.

*** *P* < 0.001 between groups.

**Fig. 2:**
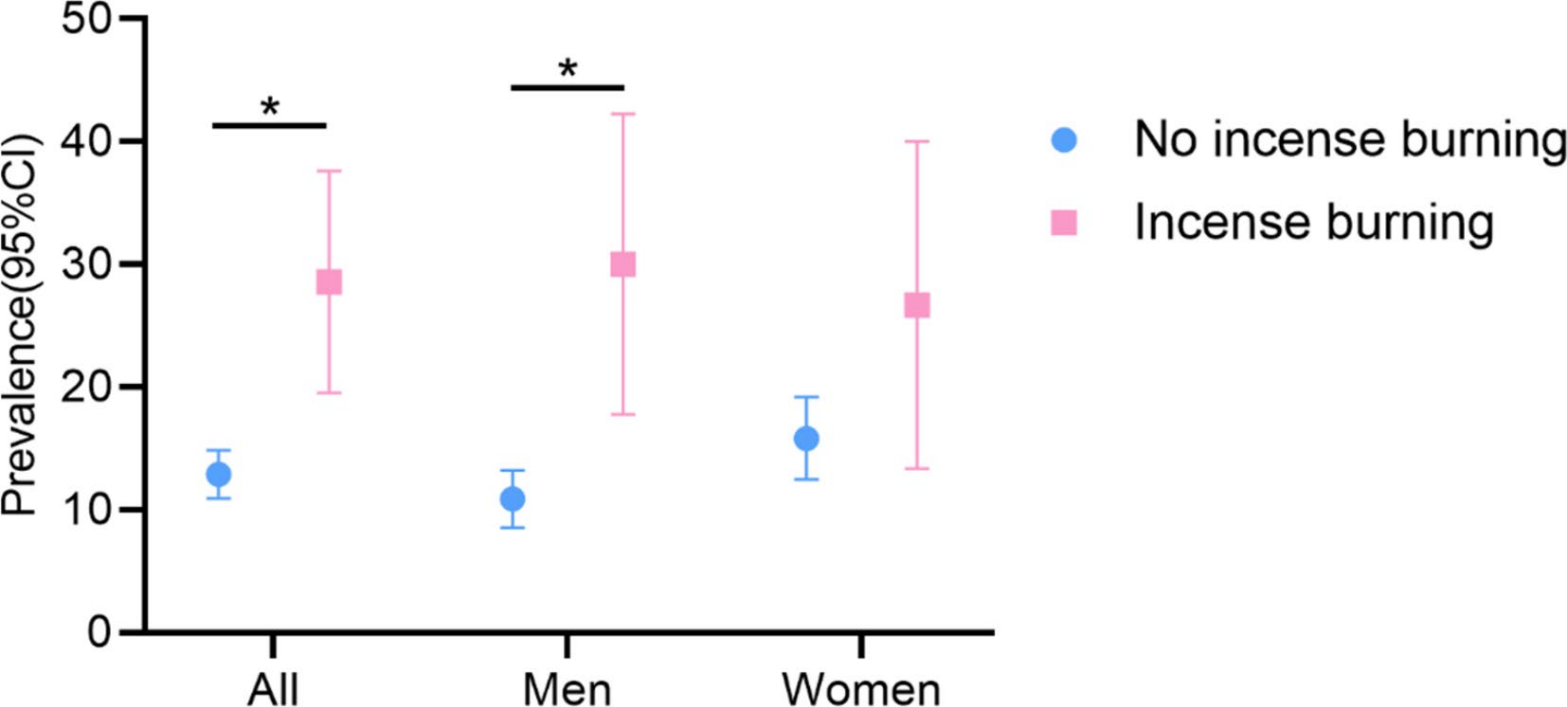
Prevalence of impaired lung function among participants, stratified by gender. 95%CI, 95% confidence interval; * *P* < 0.05 between groups.

Among the participants, 54 (14.4%) were identified as having impaired lung function, with 28 men and 26 women affected (Table 2). The prevalence of impaired lung function was notably higher in the exposed group at 28.6%, in comparison to 12.9% in the non-exposed group (*P* = 0.02; Table 2; Fig. 2). This difference was statistically significant in men (*P* = 0.03) but not in women (*P* = 0.28), as revealed by gender-stratified analysis. Specifically, for men, the prevalence of impaired lung function was 30.0% in the exposed group and 10.9% in the non-exposed group.

**Table 2:** Prevalence of impaired lung function among participants, stratified by gender.

	No Incense Burning			Incense Burning			*P* for comparison
	*n* ^a^	*N* ^b^	Prevalence, %	*n* ^a^	*N* ^b^	Prevalence, %	
All	44	341	12.9	10	35	28.6	0.02*
Men	22	202	10.9	6	20	30.0	0.03*
Women	22	139	15.8	4	15	26.7	0.28

*P < 0.05 between groups.

^a^The number of participants with impaired lung function.

^b^The number of participants in the exposed group and the non-exposed group.

The findings from the multivariable logistic regression analysis are presented in Table 3. Participants exposed to indoor incense burning were found to have a 145% higher odds of impaired lung function compared to those not exposed, after adjusting for gender and age (OR = 2.45; 95%CI: 1.04, 5.42; *P* = 0.03; Table 3). Further adjustment for gender, age, BMI, educational attainment, and smoking status resulted in an odds ratio of 2.3 (95%CI: 0.97, 5.16; *P* = 0.05). Particularly in men, exposure to indoor incense burning was associated with a higher prevalence of impaired lung function after adjusting for potential confounders (OR = 3.39; 95%CI: 1.07, 9.82; *P* = 0.03; Table 3). However, the magnitude of the association between indoor incense burning and impaired lung function in women was smaller and had wider confidence interval (OR = 1.42, 95% CI: 0.35, 4.83, Table 3).

**Table 3:** Association between indoor incense burning and impaired lung function among Chinese adults with diabetes, stratified by gender.

	All		Men		Women	
	OR(95%CI)	*P*	OR (95%CI)	*P*	OR (95%CI)	*P*
Model1	2.45 (1.04, 5.42)	0.03*	3.4 (1.1, 9.53)	0.02	1.58 (0.39, 5.28)	0.48
Model2	2.3 (0.97, 5.15)	0.05	3.36 (1.07, 9.63)	0.03*	1.41 (0.35, 4.8)	0.60
Model3	2.3 (0.97, 5.16)	0.05	3.39 (1.07, 9.82)	0.03*	1.42 (0.35, 4.83)	0.59

Model1: Adjusted for gender (not in gender-specific analysis) and age.

Model2: Adjusted for gender (not in gender-specific analysis), age, and body mass index.

Model3: Adjusted for gender (not in gender-specific analysis), age, body mass index, educational attainment and smoke status.

OR, odds ratio; CI, confidence interval.

*P < 0.05 between groups.

## Discussion

The cross-sectional study conducted in patients with diabetes uncovered a noteworthy correlation between indoor incense burning and impaired lung function after controlling for gender and age. However, the strength of the association was attenuated, and the significance was reduced after further adjusting for BMI, educational attainment, and smoking status. This investigation represents the inaugural epidemiological study to delve into this relationship specifically within the context of diabetes. Moreover, gender-specific differences in the association between indoor incense burning and impaired lung function were observed, adding an additional layer of complexity to the findings.

The findings of this study align with prior research that has demonstrated correlations between incense burning and impaired lung function across various demographic groups, encompassing adolescents and adults^21,26^. Notably, a community-based study in Saudi Arabia, which recruited 50 workers with a history of at least one year in bakhour shops, revealed a significantly heightened prevalence of impaired lung function among individuals with extended bakhour shop tenure (OR = 1.72). Furthermore, regular use of incense at home was also associated with an elevated likelihood of impaired lung function (OR = 2.05)^26^. Additional investigations have reported a spectrum of respiratory ailments in individuals with frequent exposure to incense burning^17,27^. For instance, data from adolescent asthma screenings indicated that daily exposure to incense burning was linked to diminished lung function, as evidenced by lower mean FVC and FEV_1_ measures among adolescents with daily exposure compared to those without such exposure (*P* < 0.05)^28^. Moreover, incense burning has been linked to a higher prevalence of respiratory symptoms in children, including bronchitis (OR = 1.39), bronchiolitis (OR = 1.72), asthma (OR = 1.43), and wheezing (OR = 1.49)^12,28^. While certain earlier studies have reported no significant association between burning incense and lung function, it is imperative to consider factors such as study duration that may influence the results^29^. For example, studies with limited observation periods may not fully capture the impact of incense burning on respiratory health. Additionally, it is noteworthy that no significant association was found between lung function impairment and exposure to incense burning twice a month^30^. These collective findings underscore the importance of considering various factors and nuances when evaluating the potential impact of incense burning on respiratory health across diverse populations.

This study revealed that gender played a crucial role in modifying the association between indoor incense burning and lung function impairment, with a notably stronger association observed in men compared to women. Although the number of studies in this area is limited, existing research has consistently demonstrated similar gender-specific results. For instance, a study involving 2203 Chinese children found that exposure to incense burning was linked to an increased risk of chronic cough in boys but not in girls^31^. Similarly, another study in China reported that the adverse respiratory health effects of incense burning were more pronounced in boys than in girls, as evidenced by significant associations with incense burning in boys for outcomes such as maximum mid-expiratory flow, bronchitis, bronchiolitis, pneumonia, and wheezing^12^. The modified effect of gender observed in our study may be attributed to specific biological differences, varying exposure levels, or other systematic gender-related disparities that were not fully elucidated within the scope of our study designs. Collectively, these findings underscore the significance of considering gender differences when evaluating the potential health implications of indoor incense burning.

While the precise biological mechanisms underlying the relationship between indoor incense burning and impaired lung function remain unclear, especially in adults with diabetes, several plausible pathways can be proposed based on existing evidence. Incense burning emits a complex mixture of pollutants, including fine and ultrafine particulate matter, carbon monoxide, carbon dioxide, nitrogen oxides, volatile organic compounds, heavy metals, and other gaseous compounds^20,32,33^. Elevated indoor levels of carbon dioxide and volatile organic compounds have been associated with heightened oxidative stress, which may weaken lung defense mechanisms and contribute to impaired lung function^33–35^. Additionally, studies have demonstrated that exposure to incense smoke can generate reactive oxygen species, which disrupt tight junction proteins in the bronchial epithelium and impair epithelial barrier function via the epidermal growth factor receptor-extracellular signal-regulated kinase 1/2 (EGFR-ERK1/2) signaling pathway^36,37^. Experimental studies provide further evidence of the potential pathophysiological effects of incense smoke. In one study, rats exposed to Arabian incense for 14 weeks exhibited significant lung tissue changes, including pneumocyte degeneration, necrosis, neutrophil infiltration, and thickening of alveolar walls due to collagen-fibril deposits, ultimately compromising respiratory function^38^. Similarly, mice exposed to incense smoke showed acute disruption of epithelial barrier function after a single exposure, while prolonged exposure led to peri-bronchial fibrosis, alveolar wall thickening, and lymphoid cell aggregation in the lungs^39,40^. These findings were corroborated by subsequent studies, which confirmed fibrosis and inflammatory responses in incense-exposed animals^35,41^. Given the susceptibility of individuals with diabetes to oxidative stress and inflammation^6^, it is plausible that incense smoke exacerbates pre-existing metabolic and vascular dysfunction. Chronic hyperglycemia results in the accumulation of glycosylated proteins, which promote inflammation and impair pulmonary defenses. The oxidative stress induced by incense smoke pollutants may amplify these processes, causing further damage to the alveolar-capillary membrane and impairing gas exchange. Additionally, incense-related reactive oxygen species might interact with diabetes-induced collagen glycosylation, aggravating fibrosis and restricting lung expansion. These compounded effects could render diabetic individuals particularly vulnerable to the respiratory impacts of incense smoke exposure.

It is essential to acknowledge the potential biases and limitations in our study. First of all, a small sample size leads to a wide confidence interval in this study. Although we made efforts to include a diverse range of participants, the study may still be subject to selection bias. Additionally, the use of questionnaires to obtain incense burning and covariate data introduces the possibility of information bias. Furthermore, while we sought to control for potential confounders, it is evitable that residential confounders may have influenced our findings. Moreover, due to the cross-sectional nature of our study, caution is warranted in inferring a causal relationship between incense burning and impaired lung function. Future research employing a longitudinal design and a larger sample size could enhance the validity of our findings. Despite the above limitations, this study possesses notable strengths and stands out in its field. To our knowledge, impaired lung function among adults with diabetes due to incense burning has not been previously reported.

## Conclusions

Our study contributes valuable evidence demonstrating a link between indoor incense burning and impaired lung function in patients with diabetes, irrespective of potential confounding factors. Furthermore, our findings reveal that gender plays a modifying role in the association between indoor incense burning and lung function. Given the widespread use of incense burning in various Asia and Arabian regions, the public health implications of our results are substantial. This emphasizes the urgent need for further research and the development of preventive strategies to mitigate the adverse effects of indoor incense burning on respiratory health. These findings underscore the importance of addressing this issue to safeguard the well-being of individuals exposed to indoor incense burning, particularly in populations with existing health conditions such as diabetes.

## Data Availability

The datasets used during the current study are available from the corresponding author on reasonable request.
